# Reducing the psychosocial impact of aphasia on mood and quality of life in people with aphasia and the impact of caregiving in family members through the Aphasia Action Success Knowledge (Aphasia ASK) program: study protocol for a randomized controlled trial

**DOI:** 10.1186/s13063-016-1257-9

**Published:** 2016-03-22

**Authors:** Linda Worrall, Brooke Ryan, Kyla Hudson, Ian Kneebone, Nina Simmons-Mackie, Asaduzzaman Khan, Tammy Hoffmann, Emma Power, Leanne Togher, Miranda Rose

**Affiliations:** National Health and Medical Research Council Centre for Clinical Research Excellence in Aphasia Rehabilitation (NHMRC CCRE-Aphasia), Brisbane, Australia; School of Health and Rehabilitation Sciences, The University of Queensland, Brisbane, Australia; Discipline of Clinical Psychology, University of Technology, Sydney, Australia; Southeastern Louisiana University, Hammond, Louisiana USA; Centre for Research in Evidence-Based Practice, Bond University, Gold Coast, Australia; Speech Pathology, Faculty of Health Sciences, The University of Sydney, Sydney, Australia; School of Allied Health, La Trobe University, Melbourne, Australia

**Keywords:** Aphasia, Cluster randomized controlled trial, Mood, Depression, Quality of life, Rehabilitation, Prevention

## Abstract

**Background:**

People with aphasia and their family members are at high risk of experiencing post stroke depression. The impact of early interventions on mood and quality of life for people with aphasia is unknown.

**Methods/design:**

This study will determine whether an early intervention for both the person with aphasia after stroke and their family members leads to better mood and quality of life outcomes for people with aphasia, and less caregiver burden and better mental health for their family members. This is a multicenter, cluster-randomized controlled trial. Clusters, which are represented by Health Service Districts, will be randomized to the experimental intervention (Aphasia Action Success Knowledge Program) or an attention control (Secondary Stroke Prevention Information Program). People with aphasia and their family members will be blinded to the study design and treatment allocation (that is, will not know there are two arms to the study). Both arms of the study will receive usual care in addition to either the experimental or the attention control intervention. A total of 344 people with aphasia and their family members will be recruited. Considering a cluster size of 20, the required sample size can be achieved from 18 clusters. However, 20 clusters will be recruited to account for the potential of cluster attrition during the study. Primary outcome measures will be mood and quality of life of people with aphasia at 12 months post stroke. Secondary measures will be family member outcomes assessing the impact of caregiving and mental health, and self-reported stroke risk-related behaviors of people with aphasia.

**Discussion:**

This is the first known program tailored for people with aphasia and their family members that aims to prevent depression in people with aphasia by providing intervention early after the stroke.

**Trial registration:**

This trial is registered in the Australian New Zealand Clinical Trials Registry (ANZCTR) as ACTRN12614000979651. Date registered: 11 September 2014.

## Background

The prevalence of depression after stroke is approximately 31 % [1]. Post stroke depression results in poorer functional recovery [2], higher mortality [3], and greater healthcare utilization [4]. An investment in psychological care after stroke has been estimated to lead to a reduction in health and social care costs of 37 % over 2 years [4].

Aphasia is prevalent in 31 % of first-time strokes and is still present in 60 % of these individuals at 12 months postonset [5]. Significant and life-altering psychosocial consequences, including poor vocational outcomes, changes in relationships, and social isolation, are associated with the presence of aphasia [5–8]. The incidence of depression after aphasia is estimated to be 62 % to 70 % and is higher than in stroke survivors who do not have aphasia [9]. Family members of patients with aphasia are also prone to develop depression and experience a variety of psychosocial consequences after the onset of aphasia [10, 11].

People with aphasia report difficulty accessing existing mental health services, intervention programs, or support groups that can meet their needs because of their communication disability [12]. A Cochrane review [13] reports a small effect size when treating post stroke depression with antidepressants (with cautions about side effects), but not the prevention of depression by pharmacological means. Notably, psychological approaches have shown promise for prevention [14]. However, the Cochrane prevention review could not extend recommendations to patients with aphasia since it noted that too few studies included participants with this condition.

In recognition of the need to improve longer-term outcomes in mood and quality of life for people with aphasia, an intervention program called the Aphasia Action Success Knowledge (Aphasia ASK) program has been developed. Findings from a Phase 1 feasibility study suggested the Aphasia ASK program had positive initial outcomes for people with aphasia and their family members. The Aphasia ASK program now requires evaluation on a larger scale.

### Aim

The study aim is to determine whether an early intervention, Aphasia ASK, for the person with aphasia after stroke and their family members leads to better mood and quality of life outcomes for people with aphasia, less caregiver burden, and better mental health for family members compared to an attention-control intervention (Secondary Stroke Prevention Information Program; SSPIP) at 12 months post stroke.

### Hypotheses

#### Primary hypothesis

People with aphasia who receive the Aphasia ASK intervention will have significantly better outcomes in mood [15] and quality of life [16] at 12 months post stroke compared to those who receive the attention control intervention (SSPIP).

#### Secondary hypothesis

Family members of people with aphasia who receive the Aphasia ASK intervention will have significantly better outcomes on measures of impact of caregiving [17] and mental health [18] at 12 months post stroke than family members who receive the attention control intervention (SSPIP).

In addition, people with aphasia who have completed the attention control intervention (SSPIP) are hypothesized to have a significantly better score on a stroke risk-related behavior measure [19] compared to those who receive the Aphasia ASK intervention.

## Methods

### Design

This is a multicenter cluster-randomized controlled trial. Clusters, which are represented by Health Service Districts, will be randomized to either the experimental intervention or an attention control intervention, with an equal number of Health Service Districts in each arm. Usual care will continue to the intervention and control arms. The usual care provided will be at the discretion of the hospital site and their treatment protocols. Usual care is typically considered as 1 to 2 sessions of aphasia therapy per week in addition to any required rehabilitation for other stroke-related impairments. Documentation of usual care aphasia therapy will occur. Eligible individuals with aphasia and their family members will be invited to participate upon referral to speech-language pathology services, commencing intervention as early as possible in rehabilitation, but no later than 6 months post stroke. Assessments will occur at baseline and at 12 months post stroke.

The Consolidated Standards of Reporting Trials (CONSORT) 2010 extension statement for cluster-randomized trials [20] has been used to guide the research plan. The Standard Protocol Items: Recommendations for Interventional Trials (SPIRIT) 2013 statement [21] has been used to develop the trial protocol. The Template for Intervention Description and Replication (TIDieR) guide [22] has been used to guide the description of the study interventions.

### Ethics approval

The trial sponsor is the University of Queensland funded by a National Health and Medical Research Council Project Grant from 2014-2018 [APP1060673]. The study protocol was approved by the Darling Downs Hospital and Health Service Human Research Ethics Committee in Queensland, Australia. Expedited approval for the study was also granted by the University of Queensland on the basis of approval from the Darling Downs Hospital and Health Service Human Research Ethics Committee. Amendments to the protocol during the study period will be submitted to the Darling Downs Hospital and Health Service Human Research Ethics Committee. Informed written consent will be obtained from all participants before inclusion in the trial.

### Clusters

Clusters for the study will be Health Service Districts that offer speech pathology rehabilitation services as a single operational unit across multiple site locations within a defined service area in Australia. Health Service Districts were chosen as clusters (instead of individual hospital/health service sites) to reduce the risk of treatment contamination issues that may arise from conducting both arms of the study within the same service area. Health Service Districts were also chosen so that the study interventions can be provided alongside the usual continuum of care for the study duration (that is, across multiple health facilities within the first year post stroke). An inclusion criterion for clusters is that they must provide aphasia rehabilitation services, with the capacity to provide services over the period of intervention. Clusters will be excluded if they are participating in other clinical trials at the time of randomization, which will limit the recruitment capacity and/or conflict with the intervention requirements of the current trial. Each Health Service District will provide either the experimental or the attention control intervention to a maximum of 20 people with aphasia plus their family members.

### Participants: inclusion and exclusion criteria

Participants will be people with aphasia who are within the first 6 months following a stroke and their family member(s). The diagnosis of aphasia will be based on a qualified speech pathologist’s administration of the Western Aphasia Battery-Revised [23] and clinical judgement of a qualified speech pathologist. Potential people with aphasia and their family members will be included if they are older than 18 years of age, have sufficient English language to participate without a translator, and have adequate hearing and vision levels to participate as judged by the treating speech pathologist. People with aphasia and their family member(s) will be excluded if they have concomitant progressive neurological conditions (for example, dementia) or a concurrent medical condition impacting on their mental health (for example, cancer) as confirmed by self-report. There are no other inclusion or exclusion criteria for family member participants. People with aphasia must present with their first incidence of post stroke aphasia and will be excluded for the following reasons: 1) aphasia as an etiology other than stroke, 2) a history of recurrent depression (that is, three or more previous diagnosed episodes defined as needing to see a health practitioner for treatment - either psychotherapy or medication prescribed, confirmed by self-report), 3) a current psychiatric diagnosis (for example, depressive disorder or anxiety disorder confirmed by medical record), 4) current depressive symptoms upon screening with the Stroke Aphasic Depression Questionnaire Hospital Version-10 [17] (score of 9 or more) or The Depression Intensity Scale Circles [24] (score of 3 or more), 5) receiving treatment in a psychiatric setting, or 6) enrolled in other aphasia or depression treatment studies.

### Process of screening and seeking consent

Potential participants with aphasia will be identified by a member of the rehabilitation team. Speech pathologists involved in the study will check the potential participant for eligibility based on the inclusion/exclusion criteria. Each potential participant with aphasia will be provided with a written participant information sheet and consent form and will be given a verbal explanation of the research. The participant’s capacity to decide whether to participate will be judged by the speech pathologist (trained in communicating with and verifying comprehension of adults with aphasia). Once participants with aphasia have given consent, they will be asked if they would like a family member to be involved in the study and to nominate this family member. If the family member agrees, the speech pathologist will verbally explain the study as well as provide family members with a participant information sheet and obtain their written informed consent.

### Randomization

A factor that may affect the outcome of this study is the level of psychological services provided to patients in each cluster. A National Stroke Foundation Rehabilitation Services Report (2012) indicated that lower levels of psychological care might be provided to patients with stroke in nonurban areas in Australia. Hence, clusters will be selected in a way such that they represent either urban (capital city) or nonurban (regional/rural) areas. Considering a cluster size of 20, the required sample size of *N* = 344 (see sample size estimates for details) can be achieved from 18 clusters (nine per arm, with five in urban and four in nonurban clusters). However, 20 clusters will be recruited to account for the potential of cluster attrition during the study. See Table 1 for how the 20 clusters will be stratified. Randomization will occur using a computer-generated random number scheme. Sequential numbers will be assigned to each cluster within each stratum, and only the cluster numbers will be sent to the trial statistician to ensure allocation concealment.

**Table 1:** Stratification of clusters

	Experimental intervention	Attention control intervention
Urban clusters (*n* = 10)	five clusters	five clusters
Nonurban clusters (*n* = 10)	five clusters	five clusters

### Intervention details

Characteristics of both the experimental and attention control interventions are summarized in Table 2. The attention control arm of the study (SSPIP) will be provided in a similar dosage and format to the experimental intervention. The SSPIP condition will control for the attention and time provided by speech pathologists to participants [25]. The provision of secondary stroke prevention information has had no known demonstrated effect on the primary outcomes in this study. All study treatments provided to participants will be documented (that is, number of sessions of the intervention completed and duration of each session). Both interventions will be provided in addition to usual care. Documentation of usual care will occur during the treatment period and include, at a minimum 1) the hours and type of speech-language pathology aphasia services received, 2) the hours of counselling provided by health professionals (for example, social worker or psychologist), and 3) stroke- or aphasia-related support group attendance.

**Table 2:** Characteristics of the experimental and the attention control interventions

	ARM 1: Experimental intervention	ARM 2: Attention control intervention
	Aphasia Action Success Knowledge (Aphasia ASK)	Secondary Stroke Prevention Information Program (SSPIP)
Participants	People with aphasia and their family members	
Timing of intervention	Participants will commence the intervention at any time before 6 months post stroke. Intervention will commence within 14 days of baseline assessments and will be completed at 12 months post stroke.	
Intervention delivery mode	Intervention modules delivered face to face in 1:1 sessions.	
	Follow-up sessions conducted over the telephone.	
Duration of intervention	Face-to-face intervention: 6 weeks of intervention modules delivered 1 module/week for 1 to 2 hours (minimum dosage = 3 modules completed and total contact time of 3 hours).	
	Follow-up phone intervention: up to 9 months of follow-up phone calls (of ½ to 1 hour duration) delivered monthly until 12 months post stroke (minimum dosage = 4 phone calls completed and total time of 2 hours).	
Intervention provider	Qualified speech-language pathologist trained by the research team in either ARM 1 or ARM 2 interventions.	
Intervention provider training	Therapy manuals provided to therapists, mandatory completion of literature readings, and mandatory completion of either face-to-face or online workshop for approximately 6 hours. If online training occurs it will be recorded and accessed via Adobe Connect software.	
Setting of intervention delivery	The intervention will be provided on site at the health service where the participant is receiving rehabilitation or, if the patient has been discharged from rehabilitation services, in the participant’s home.	
Intervention process	Therapists will guide participants through the modules, setting goals, discussing content and answering participant questions and/or concerns. Tailoring of the content will occur in that participants will select the modules they would like to complete and in which order. Tailoring will also occur for differing levels of aphasia severity to ensure the intervention is communicatively accessible (for example, using conversational support strategies and seeing people with more severe aphasia in person rather than conducting the session over telephone for the follow-up).	
Intervention content	six modules covering the following themes:	six modules covering the following themes:
	• aphasia and stroke education	• stroke education
	• basic communication strategies	• risk factor education
	• strategies for managing mood	• lifestyle modifications for managing stroke risk factors
	• strategies for maintaining social network support	• medications for stroke prevention
Intervention materials	Written support materials for each module with modifications made to formatting (for example, larger fonts and bolding of key words) in order to improve accessibility of information for people with aphasia. Additional video materials will be made available for some modules. Materials will be made available after the completion of the trial.	
Fidelity of treatment	All treatment sessions will be video recorded. The first module completed for all participants will be submitted to research team for evaluation of patient interaction skills and content delivered. If deviation of fidelity is observed retraining of the therapist will occur before administration of the intervention continues. Following all first session evaluations, a random sample of videos will be selected to check fidelity is maintained throughout the study.	

### Primary outcomes

The two primary outcomes for people with aphasia will be mood as measured by the Stroke Aphasic Depression Questionnaire- 21 item (SADQ-21) [15] and quality of life as measured by the Assessment for Living with Aphasia (ALA) [16].

### Secondary outcomes

The secondary outcome for people with aphasia will include a 10-item measure of self-reported stroke risk-related behaviors [19]. Both ideal (for example, taking medication as prescribed) and nonideal behaviors (for example, smoking cigarettes) will be measured with higher scores out of 10 indicating performance of more ideal behaviors. Secondary outcomes for family members of people with aphasia will be the impact of caregiving measured by the Bakas Caregiving Outcomes Scale Revised (BCOS) [17] and mental health as measured by the General Health Questionnaire-28 item (GHQ) [18].

### Blinding

People with aphasia, their family members, and outcome assessors will be blinded to the study design and treatment allocation (that is, will not know there are two arms to the study). The speech-language pathologist who completes the outcome assessment will be different from the treating therapists. Unblinding of the outcome assessors will not result in the discontinuation of a participant’s involvement in the study. Attempts will be made to replace the outcome assessor if unblinding occurs and re-administration of any unblinded assessments will occur.

### Data management and monitoring body

Data will be collected and managed using REDCap, an Internet-based data capture tool designed for research studies. Clinicians from each hospital site will enter participant data directly into REDCap. Once data collection has commenced, data will be monitored for completeness and accuracy by the study’s trial manager. The study’s chief investigators will monitor study progress and adverse safety events as well as audit data accuracy on an ongoing basis. No formal criteria exist for discontinuing the trial early.

### Sample size estimates

Sample size calculations were calculated for both primary outcome measures (ALA and SADQ-21). Power calculations on the ALA have been calculated from an intensive aphasia treatment study [26] and an Australian longitudinal aphasia study [27]. Power calculations on the SADQ have been calculated from the Cost analysis of the Communication and Low Mood (CALM) study [28]. The ALA required a larger sample size compared to the SADQ-21, and therefore, the larger sample size required by the ALA was determined necessary to adequately power the study. To achieve a power of 80 % with a 5 % level of significance in comparing the two arms of the study (Aphasia ASK versus attention control - SSPIP), we need 186 patients (93 per arm) with an effect size of 0.367, computed using ALA data (26, 27). The extent to which power is diminished by clustering was considered in relation to the design effect (DE) = 1 + (m -1)r10, where m = the average size of a cluster and r is the intra-class correlation coefficient. Typically, intraclass correlation coefficients are small (<0.02); thus a conservatively estimated intra-class correlation of 0.02 was used. A cluster size of 20 was chosen based on the feasibility of running the intervention, as well as the availability of patients with aphasia within clusters. Thus DE = 1+ (20-1)*0.02 = 1.38, and the total sample size required was calculated as 186 * 1.38 ≈ 258. To account for an attrition rate of 25 % to the 12-month follow-up period, 344 patients would be needed (172 per arm).

### Statistical analyses

Baseline characteristics of participating patients of the two arms will be presented and compared for any meaningful differences at baseline. Outcomes of interest will be analyzed on an intention-to-treat basis. Multilevel modelling using mixed models, which takes into account patients being nested within clusters, will be able to examine whether changes in the outcomes, which are on interval-scale, vary over time as well as across the two (Aphasia ASK and attention control- SSPIP) programs, after adjusting for the effects of any potential confounders (if any). In addition, attrition patterns across the two arms will be examined to determine randomness of missing data, and if required, multiple imputations will be implemented. All statistical analyses will be performed using Stata statistical software. An alpha level of 0.05 will be accepted as significant. The results of the statistical models will be presented in the form of regression coefficients, their 95 % confidence intervals, and effect sizes. The residuals of the fitted models will be examined to ensure that all required assumptions are met. The statistician completing the data analyses will be blinded to group allocation until analysis is completed.

### Additional data collection and analyses

Qualitative interviews and/or survey data will also be collected from participants and treating speech pathologists upon completion of the 12-month evaluation and/or the end of the study. The interviews and/or survey data will gather information about the treatment services that have been provided.

## Discussion

This is the first known intervention tailored for people with aphasia that aims to prevent depression and improve longer-term outcomes by providing intervention early after stroke. The intervention is of reasonably low intensity, and if effective and integrated into speech pathology clinical practice, it has the potential to not only improve mood and quality of life but also other functional outcomes impacted by mood.

### Trial status

The target number of 20 clusters has been recruited, of which 14 have had the relevant governance application approved to start the trial. Clusters that have been approved include the Darling Downs Service District, QLD; Wide Bay Hospital and Health Service, QLD; Mackay Hospital and Health Service, QLD; Western New South Wales Local Health District, NSW; Northern Sydney Local Health District, NSW; Sacred Heart Rehabilitation Service, NSW; Hunter New England Local Health District, NSW; Western Health, VIC; Barwon Health, VIC; Northern Health, VIC; Monash Health, VIC; Peninsula Health, VIC; Tasmanian Health Service- South, TA; and The Canberra Hospital and Health Services, ACT.

## Abbreviations

ALA: Assessment for Living with Aphasia
Aphasia ASK: Aphasia Action Success Knowledge
BCOS: Bakas Caregiving Outcomes Scale revised
CONSORT: Consolidated Standards of Reporting Trials
GHQ: General Health Questionnaire
SADQ-21: Stroke Aphasic Depression Questionnaire- 21 item
SPIRIT: Standard Protocol Items, Recommendations for Interventional Trials
SSPIP: Secondary Stroke Prevention Information Program
TIDieR: Template for Intervention Description and Replication
